# Air Pollution Distribution Patterns in the San Bernardino Mountains of Southern California: a 40-Year Perspective

**DOI:** 10.1100/tsw.2007.57

**Published:** 2007-03-21

**Authors:** Andrzej Bytnerowicz, Michael Arbaugh, Susan Schilling, Witold Fraczek, Diane Alexander, Philip Dawson

**Affiliations:** ^1^ USDA Forest Service, Pacific Southwest Research Station, Riverside, CA, USA; ^2^ Environmental Systems Research Institute, Redlands, CA, USA

**Keywords:** ozone, nitric acid, ammonia, mixed conifer forest, anthropogenic pressure

## Abstract

Since the mid-1950s, native pines in the San Bernardino Mountains (SBM) in southern California have shown symptoms of decline. Initial studies in 1963 showed that ozone (O_3_) generated in the upwind Los Angeles Basin was responsible for the injury and decline of sensitive trees. Ambient O_3_ decreased significantly by the mid-1990s, resulting in decreased O_3_ injury and improved tree growth. Increased growth of trees may also be attributed to elevated atmospheric nitrogen (N) deposition. Since most of the N deposition to mixed conifer forest stands in the SBM results from dry deposition of nitric acid vapor (HNO_3_) and ammonia (NH_3_), characterization of spatial and temporal distribution of these two pollutants has become essential. Although maximum daytime O_3_ concentrations over last 40 years have significantly decreased (~3-fold), seasonal means have been reduced much less (~1.5-fold), with 2-week long means occasionally exceeding 100 ppb in the western part of the range. In the same area, significantly elevated concentrations of HNO_3_ and NH_3_, up to 17.5 and 18.5 μg/m^3^ as 2-week averages, respectively, have been determined. Elevated levels of O_3_ and increased N deposition together with long-term drought predispose the SBM forests to massive bark beetle attacks making them susceptible to catastrophic fires.

## INTRODUCTION

Since the mid-1950s, mid-elevation forests in the San Bernardino Mountains (SBM) have shown symptoms of significant decline, which was first shown on its most sensitive species, ponderosa pine (*Pinus ponderosa*). Initially, the condition was termed "x-disease" because its cause was unknown[1]. However, as a result of field and controlled experiments, in which seedlings, saplings, and branches of trees were exposed to charcoal-filtered clean air and ambient smog, it was revealed that ozone ($O_3$), an important component of smog, was the main cause of the decline[2]. Photochemical smog generated in the heavily populated Los Angeles Basin (LA Basin) is trapped by thermal inversion (up to about 2,000 m above the sea level). In a typical summer season, the polluted LA Basin air masses move inland with easterly winds[3] and are intercepted by the SBM range.

About 70% of air pollution in the LA Basin, where presently ~14 million of people live, results from combustion of gasoline and diesel fuel by ~12 million motor vehicles[4]. Population growth in southern California is the highest in the entire nation. While the U.S. population has nearly doubled since 1950, in the entire state of California it tripled, and it grew even more in southern California[5]. Historical and projected population changes for the six counties of the Southern California Association of Governments (SCAG) region indicate that growth was the highest in those counties from which emissions directly affect air pollution status of the SBM. The highest population growth between 1960 and 2000 was recorded for Riverside County (5.05-fold increase), Orange County (4.04-fold increase), and San Bernardino County (3.4-fold increase), compared with 1.58-fold growth in Los Angeles County[6].

During this rapid expansion of the southern California population, several air pollution control initiatives were initiated by the California Air Resources Board (CARB) and implemented by South Coast Air Quality Management District (SCAQMD)[7]. These measures resulted in a significant reduction in pollutant emissions as well as a large reduction of ambient $O_3$ concentrations in the LA Basin (Fig. 1). Cleaner burning fuel also delayed onset of $O_3$ formation, resulting in smaller $O_3$ decreases in more easterly mountain and desert areas than near urban areas. Recent studies[8] indicate the spatial distribution of air pollution may also have changed, but a lack of monitoring data has prevented characterization of spatial trends.

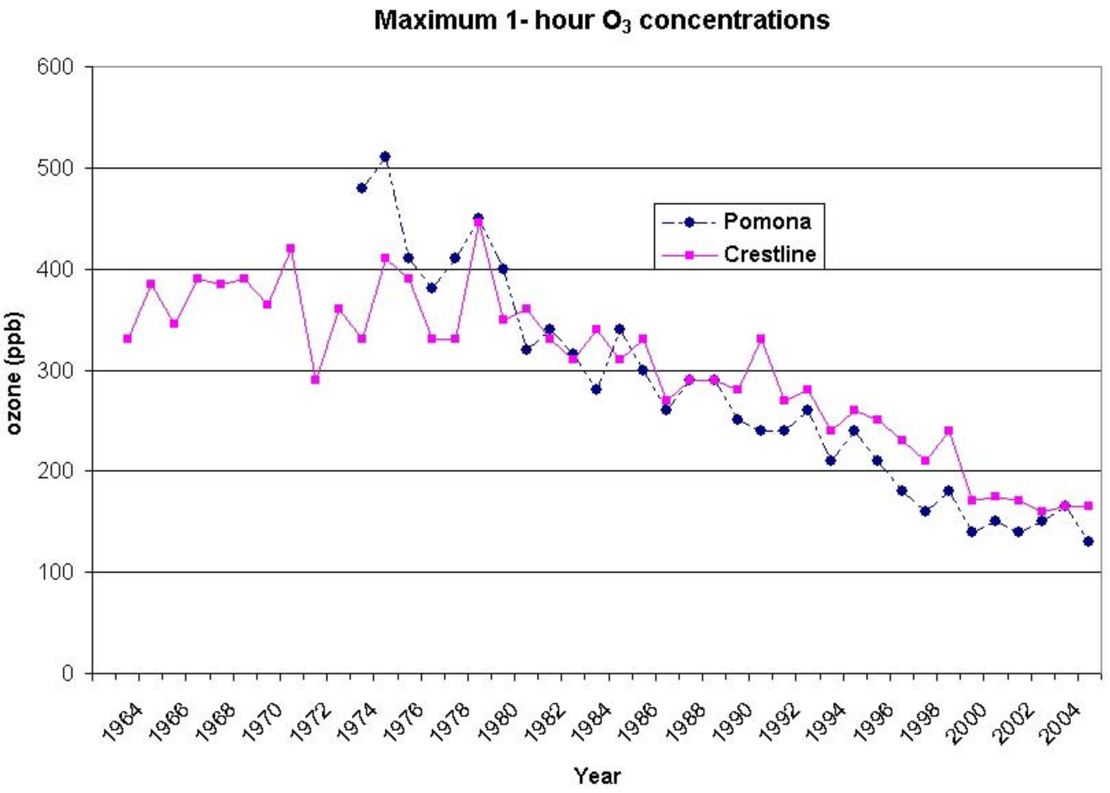

**FIGURE 1.** Changes in maximum 1-h $O_3$ concentrations per year in the South Coast Air Basin: Pomona (low elevation location in the center of the Basin) and Crestline (high elevation site on the west-exposed crest of the SBM).

Since 1990, Forest Service scientists have studied interactive effects of $O_3$, wet and dry deposited nitrogenous (N) compounds, climatic variability, water availability, and outbreaks of insects on physiological responses of trees, their growth and mortality, and changes in hydrological and soil processes in forests. The following first description of spatial trends of major air pollutants in the SBM is critical to understanding the ongoing impacts of air pollution on the highly stressed forest areas of southern California.

## MATERIALS AND METHODS

### Monitoring Network

In 2002, 18 sites were established in the SBM for air pollution and forest health monitoring (Fig. 2). These sites included 11 original sites of the 1968–1974 study[2] as well as the newly established sites[9]. The sites were selected following ground inspection and available information on distribution of photochemical smog, soil condition, forest condition, and stand/tree health[9]. Monitoring sites were selected in open-terrain locations, such as forest clearings, burnt areas, forest nurseries, etc. Sites were located on a western aspect, at least 100 m from a local road and 200 m from main roads. Free air movement from all directions was required; however, sites exposed to continuously strong winds were avoided. Sampler stands were placed at a distance at least two times the height of the tallest tree from forest edges, however, sparsely dispersed smaller trees or shrubs that did not directly obstruct the samplers were allowed. Passive samplers were exposed for 2-week periods during the summer and fall seasons of 2002–2005. Monitoring was performed between May and October/November; beginning and end of the monitoring campaigns depended on weather conditions. Samplers in protective caps were hung on a wooden stand about 2 m above ground level.

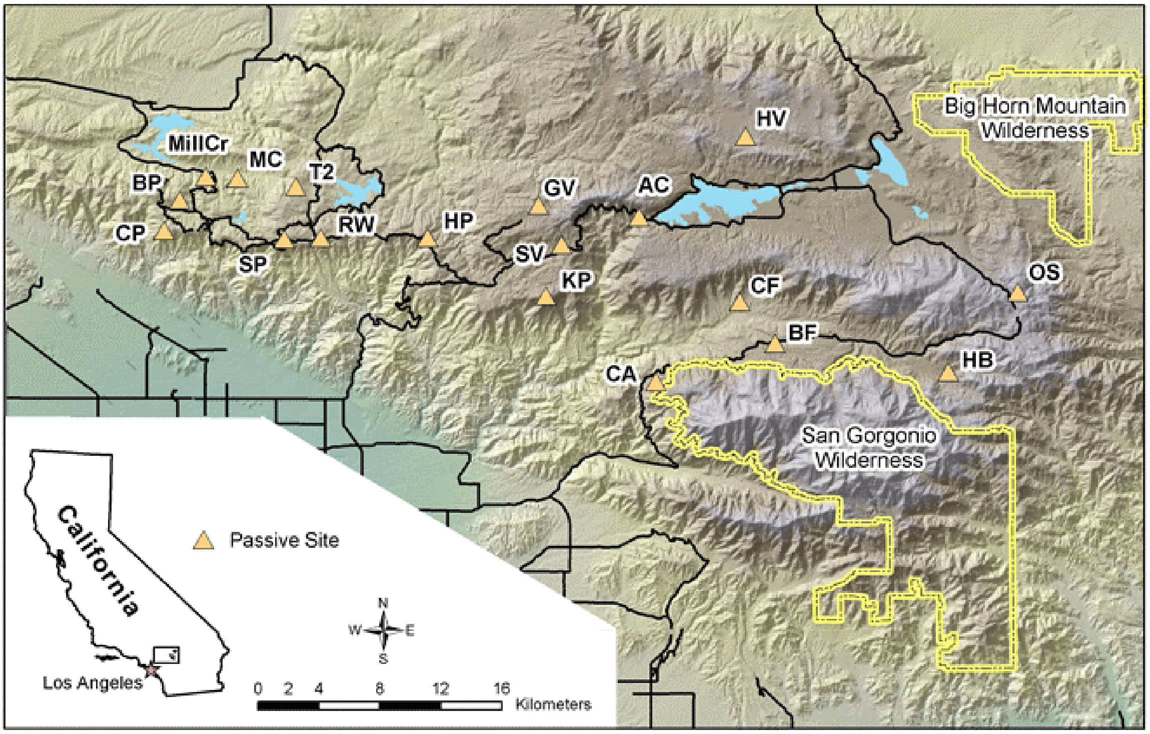

**FIGURE 2**. Locations of air pollution/forest health monitoring sites in the SBM from West to East: CP, Camp Paivika; BP, Breezy Point; MillCr, Mill Creek; SP, Strawberry Peak; MC, Miller Canyon; T2, Tunnel 2; RW, Rim of the World; HP, Heaps Peak; SV, Snow Valley; GV, Green Valley; KP, Keller's Peak; CA, Camp Angeles; AC, Arctic Circle; HV, Holcomb Valley; CF, Converse Flats; BF, Barton Flats; HB, Heart Bar; OS, Onyx Summit.

## Equipment Used

Ogawa samplers (Pompano Beach, FL) were used to measure $O_3$ concentrations[10]. In each sampler, two replicate cellulose filters saturated with nitrite ($NO_2^-$) were used. Nitrite on the filters is oxidized by ambient $O_3$ to nitrate ($NO_3^-$). Nitrate concentration in filter extracts was determined by ion chromatography (Dionex, Model 4000i) and the rate of $NO_3^-$ formation served as a measure of 2-week average ambient $O_3$ concentration at every site. Rates of $NO_3^-$ formation in the passive samplers were compared to real-time $O_3$ concentration measurements by UV absorption (2 B Technologies) and the empirically derived coefficients were used to calculate $O_3$ concentrations.

Passive samplers developed by the USDA Forest Service[11] were used for monitoring nitric acid ($HNO_3$) concentrations. In that sampler, ambient air passes through Teflon membrane and gaseous $HNO_3$ is absorbed on Nylasorb nylon filter as $NO_3^-$. Nitrate concentrations in sample extracts were analyzed by ion chromatography (Dionex, Model 4000i) and concentrations of $HNO_3$ were calculated using the calibration curves[11]. Three replicate $HNO_3$ samplers were used at each monitoring site.

Ammonia ($NH_3$) concentrations were determined with Ogawa passive samplers. Ammonia is absorbed on two replicate cellulose pads coated with citric acid forming ammonium citrate. Ammonium concentrations in filter extracts were determined colorimetrically on a Technicon Autoanalyzer. Concentrations of $NH_3$ from passive samplers were calculated based on a comparison with colocated anular denuder systems[12].

## Geostatistical Analysis

Pollutant distribution maps were developed with the Geostatistical Analyst, extension of ArcGIS software (ESRI, Redlands, CA) software. The Geostatistical Analyst uses values measured at sample points at different locations in the landscape and interpolates them into a continuous surface. Using a set of pollutant concentration measurements for the study area, a spatial model of $O_3$ concentration was developed using the inversed distance weighted (IDW) method[13].

## RESULTS

- Ozone — Seasonal average concentrations of the pollutants in 4 years of the study based on consecutive 2-week long collection periods were used in the analysis (Table 1). Ranges of $O_3$ concentrations were similar for 2002, 2003, and 2004, while in 2005, there were larger differences between the minimum and maximum values. Mean seasonal concentration was the lowest in 2003 and the highest in 2002. Consistently, the highest $O_3$ concentrations were recorded in the western part of the network closest to the LA Basin, especially at the Camp Paivika (CP), Rim of the World (RW), and Heaps Peak (HP) monitoring sites. In 2003, high levels of $O_3$ were also detected at the Keller Peak (KP) and Arctic Circle (AC) sites and at the Heart Bar (HB) site open to Banning Pass and Interstate 10. Lowest $O_3$ concentrations were found north of Big Bear Lake at the Holcomb Valley (HV) site (Fig. 3A–D).
- Nitric acid — Ranges of $HNO_3$ concentrations were lower in 2002 and 2003 than in 2004 and 2005. However, mean $HNO_3$ concentrations for 2002, 2004, and 2005 were much higher than for 2003 (Table 1). The highest levels of the pollutant were always recorded in the western part of the network. In 2002, 2004, and 2005, there was a sudden drop of $HNO_3$ concentrations east of the HP site that continued eastwards. In 2003, high concentrations of $HNO_3$ were also recorded along the Santa Ana River Valley at KP and Converse Flats (CF). Similarly to $O_3$, the lowest values of $HNO_3$ were found at the HV site north of Big Bear Lake (Fig. 4A–D).

**TABLE 1**
**Summary of Air Pollution Monitoring for the SBM Network, Based on 2-Week Long Collection Periods**

| Year | Duration | No. Sites | $O_3$ (ppb) | | $HNO_3$ (μg/m$^3$) | | $NH_3$ (μg/m$^3$) | |
|---|---|---|---|---|---|---|---|---|
| | | | Range | Mean | Range | Mean | Range | Mean |
| 2002 | 5/20–10/10 | 13 | 36.1–88.1 | 57.5 | 0.8–10.4 | 4.3 | 0.0–7.0 | 3.1 |
| 2003 | 6/12–11/20 | 17 | 25.7–78.8 | 48.8 | 0.3–12.2 | 2.8 | 0.0–18.5 | 4.4 |
| 2004 | 6/3–11/5 | 16 | 27.2–84.7 | 52.5 | 0.9–17.5 | 4.2 | 0.7–9.1 | 4.0 |
| 2005 | 5/4–10/4 | 16 | 17.7–102.6 | 55.2 | 0.5–16.8 | 3.9 | 0.0–9.8 | 3.2 |

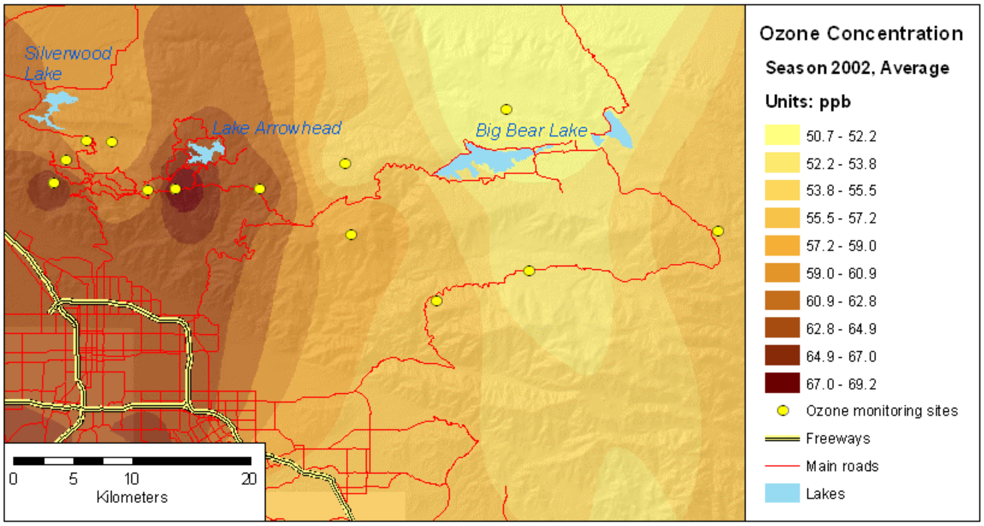

**A**

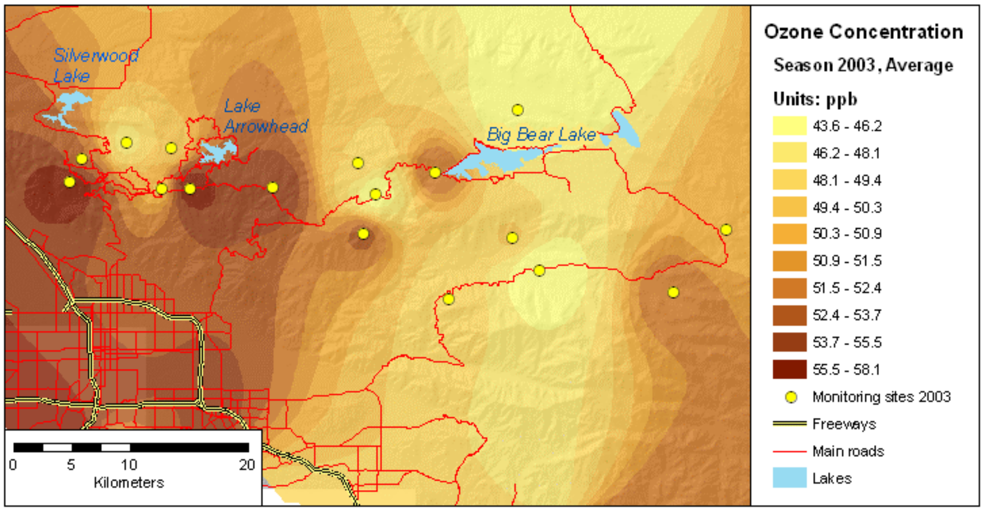

**B**

**FIGURE 3.** Distribution of $O_3$ concentrations determined with passive samplers in the SBM; (A) 2002, (B) 2003, (C) 2004, and (D) 2005.

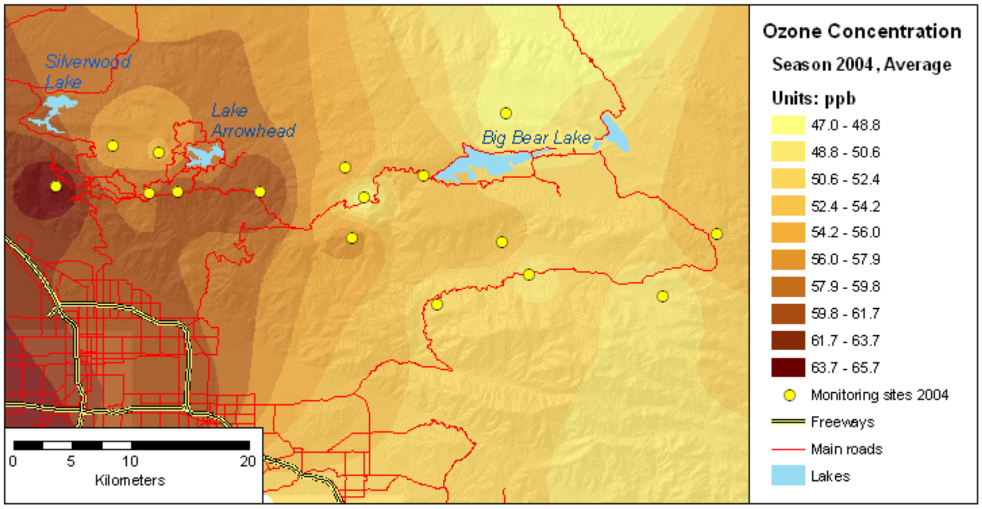

**FIGURE 3C**

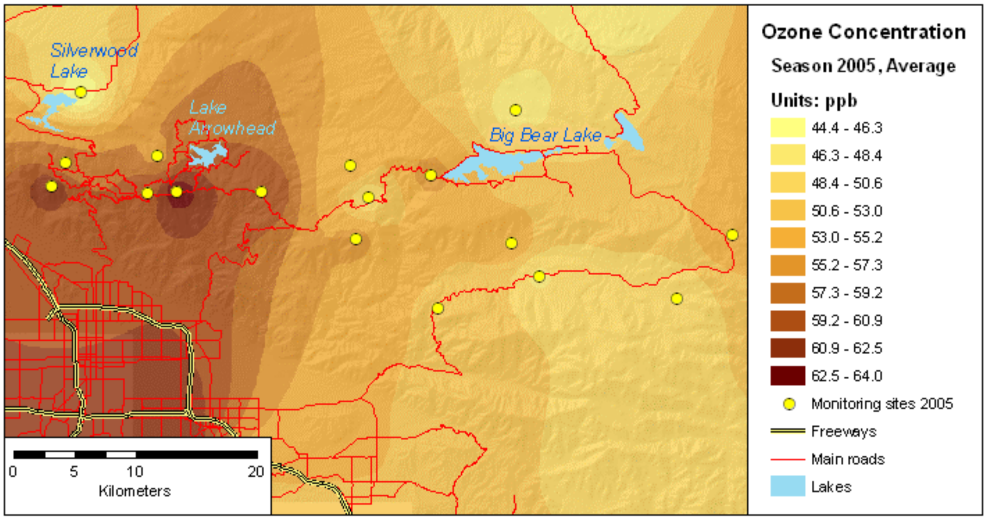

**FIGURE 3D**

- Ammonia — Ranges of $NH_3$ were similar in 2002, 2004, and 2005, while in 2003, much higher maximum values of $NH_3$ were recorded. Mean values in 2002 and 2005 were lower than in 2003 and 2004 (Table 1). Distribution of the pollutant was, in general, similar to those of $O_3$ and $HNO_3$ with the highest concentrations in the western part of the network. Consistently, the highest concentrations were recorded at the Strawberry Peak (SP) and RW sites. High concentrations of the pollutant, especially in 2002, were also found at the Camp Angeles (CA) site near the San Gorgonio Wilderness area. Lowest $NH_3$ concentrations were in the eastern part of the network near Big Bear Lake (Fig. 5A–D).

## DISCUSSION

Most available historical information regarding air pollution in the SBM is on $O_3$. Lee et al.[14] reconstructed hourly $O_3$ data for the Crestline site, which has been near the CP site of our network since

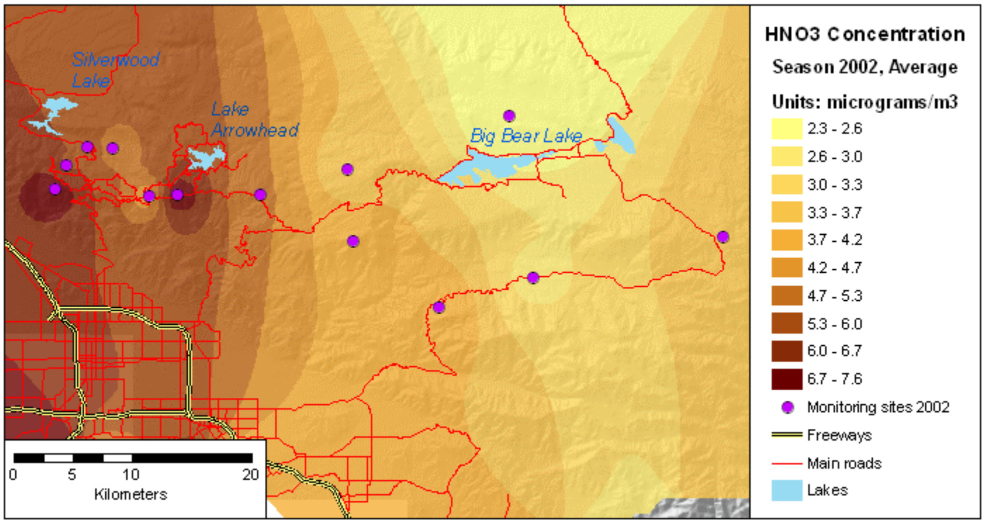

**A**

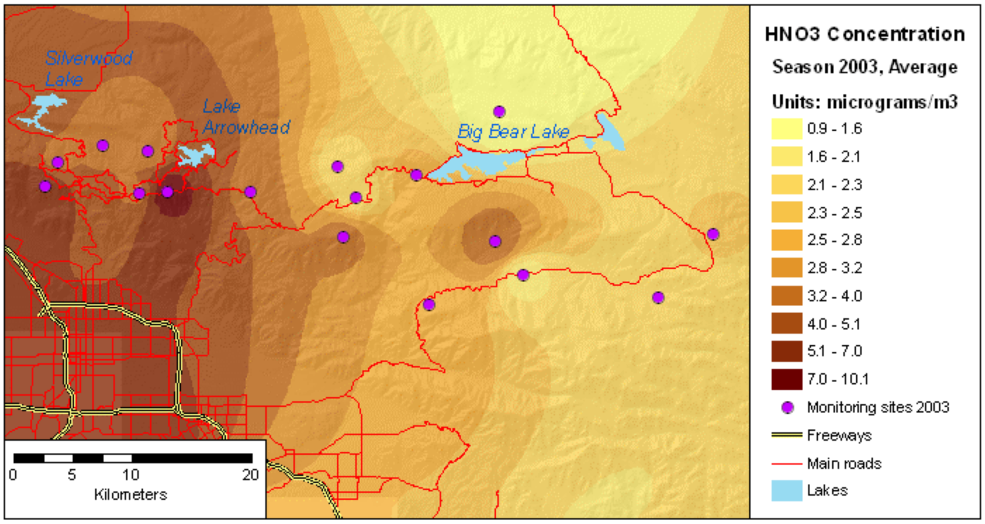

**B**

**FIGURE 4**. Distribution of $HNO_3$ concentrations determined with passive samplers in the SBM; (A) 2002, (B) 2003, (C) 2004, and (D) 2005.

1963. Mean 24-h concentrations of $O_3$ for the June–September periods obtained from the Lee et al.'s paper for 1963–2000 and for 2001–2006 from the CARB database[15] are shown in Fig. 6. While mean $O_3$ concentrations in the 1970s could reach >100 ppb, they gradually diminished, reaching a plateau oscillating around 60–70 ppb starting in 1995 and continuing until today. This is in clear contrast to the continuously decreasing hourly maximum $O_3$ concentrations at the Crestline (Fig. 1). This phenomenon of decreasing maximum $O_3$ concentrations and continuing high levels of long-term average $O_3$ concentrations is quite typical for the Northern Hemisphere, especially in remote mountainous areas[16]. In the SBM, a very clear decrease of maximum $O_3$ levels was caused by effective air pollution control measures introduced in California since the late 1970s. Reformulation of gasoline introduced in 1992 delayed $O_3$ formation further eastward, decreasing urban levels proportionally more than in the easterly

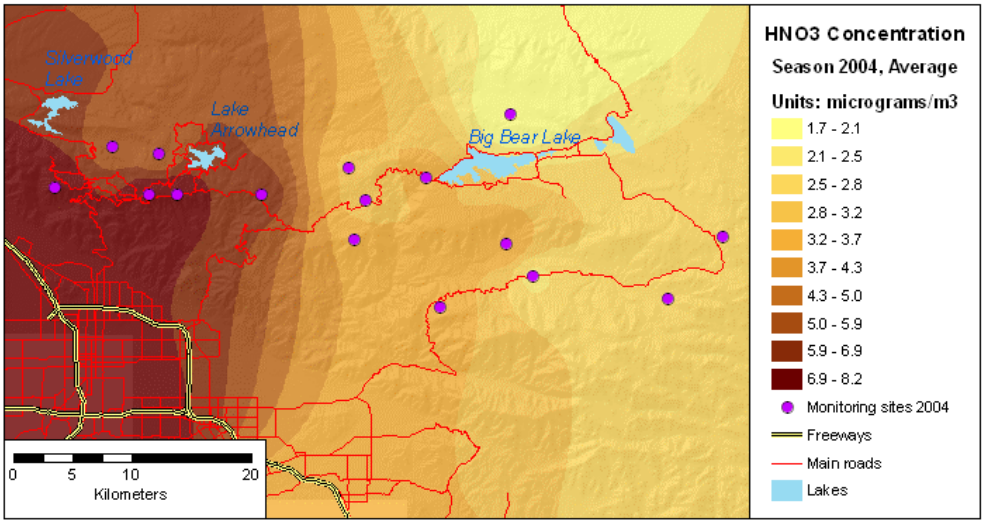

**FIGURE 4C**

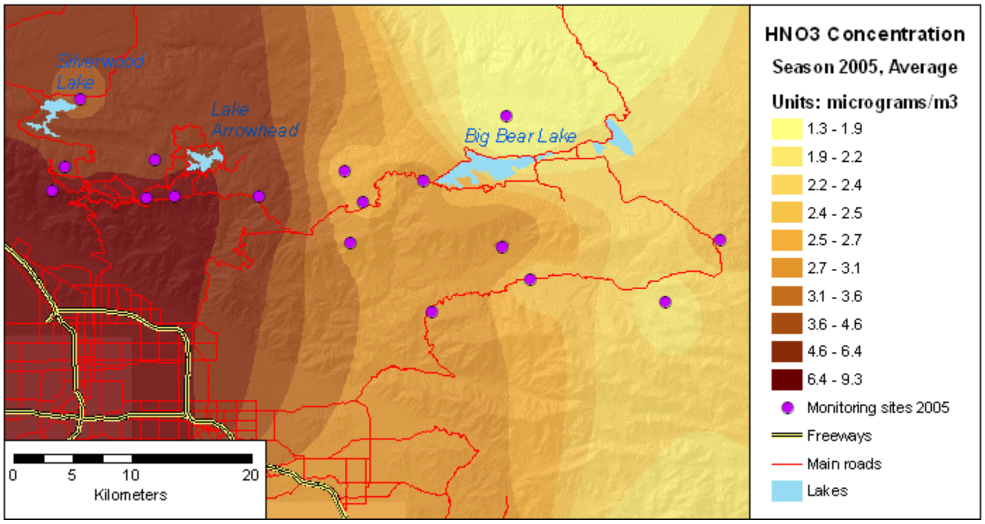

**FIGURE 4D**

valleys and mountains including the SBM. However, as urban sprawl continues and traffic intensifies in the eastern part of the LA Basin and in the Banning/Palm Springs area, $O_3$ is distributed over larger areas. This situation results in elevated 24-h mean concentrations and less pronounced west-east gradient of $O_3$ in the SBM compared to the 1970s[17]. High mean $O_3$ values in the mountain locations may be partially explained by the elevated levels of the pollutant continuing throughout the night hours due to a lack of nitric oxide (NO) that effectively scavenges $O_3$ in the urban source areas[18]. In general, our study shows that although the maximum $O_3$ concentrations have significantly decreased over 40 years of studies, high mean values occurring in the western part of the range have a strong potential for physiological and biochemical effects[19], damage of sensitive species and individuals, and predisposition of trees to drought stress and bark beetle attacks[20].

Much less is known on distribution of $HNO_3$ and $NH_3$ in the studied area. First measurements of these pollutants were performed in 1992 and 1993 at Barton Flats in the middle of the SBM air pollution gradient. Summer season mean values calculated for June–September 1991–1993 were 1.4 μg/m$^3$ for $HNO_3$ and 2.85

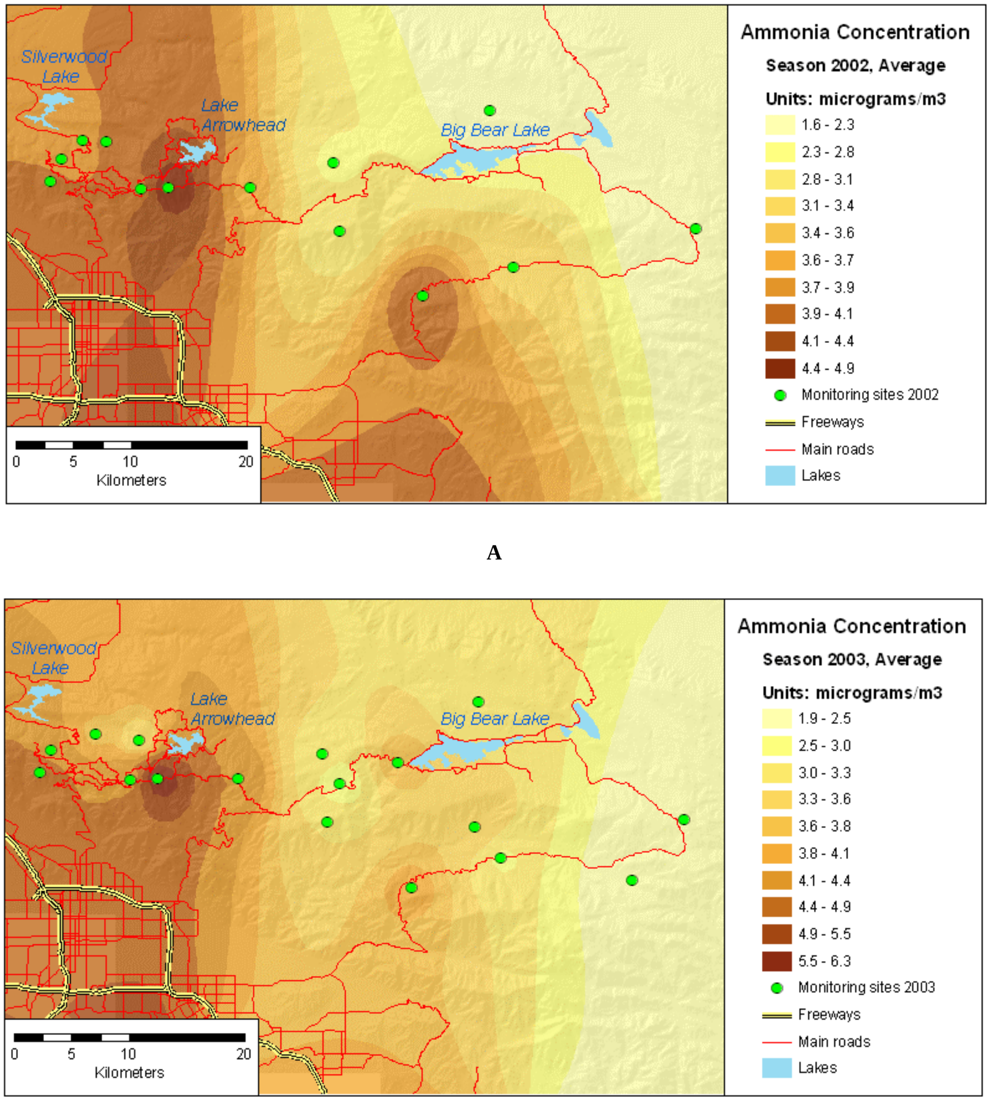

**A**

**B**

**FIGURE 5.** Distribution of $NH_3$ concentrations determined with passive samplers in the SBM; (A) 2002, (B) 2003, (C) 2004, and (D) 2005.

μg/m$^3$ for $NH_3$[21]. At same location during the 2002–2005 study, the June–August $HNO_3$ means ranged between 1.7 in 2003 to 3.2 μg/m$^3$ in 2002, with a 4-year mean of 2.5 μg/m$^3$. For $NH_3$, such values ranged between 2.8 in 2003 to 4.1 μg/m$^3$ in 2004, with a 4-year mean of 3.3 μg/m$^3$. The recorded concentrations were very high; background levels of $HNO_3$ in remote areas summer should not exceed 0.4 μg/m$^3$[22] while those for $NH_3$ should be less than 1 μg/m$^3$[23]. The observed increase in $NH_3$ concentrations may reflect effects of the reformulation of gasoline that promotes higher production of reduced N compounds from combustion engines equipped with catalytic converters[24]. Reasons for the relatively high increase in $HNO_3$ levels since 1991–1993 are not clear and will have to be further investigated. Elevated levels of $HNO_3$ and $NH_3$, especially in the western part of the range, explain why these areas experience high levels of N deposition. Both $HNO_3$ and $NH_3$ have very high deposition velocities to foliar surfaces[23], and can also be effectively taken up by stomata[25].

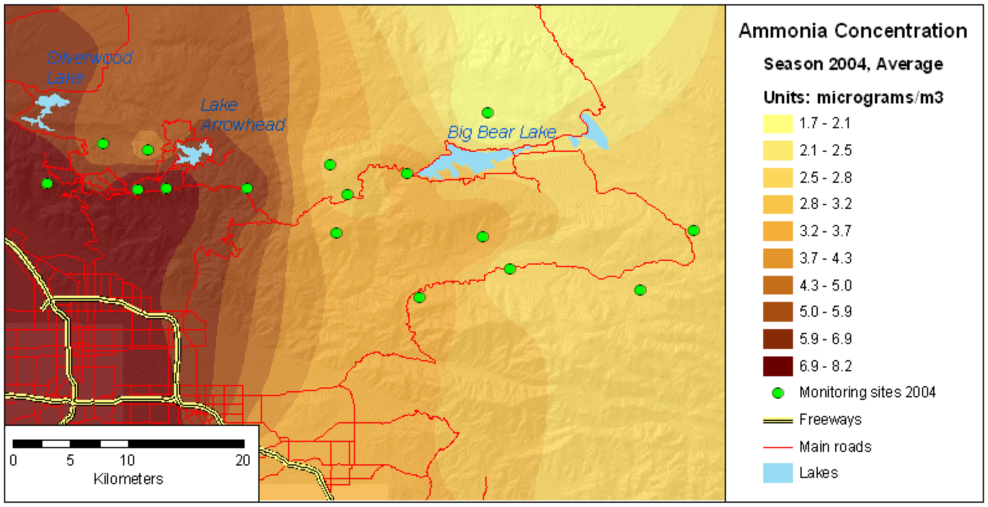

**FIGURE 5C**

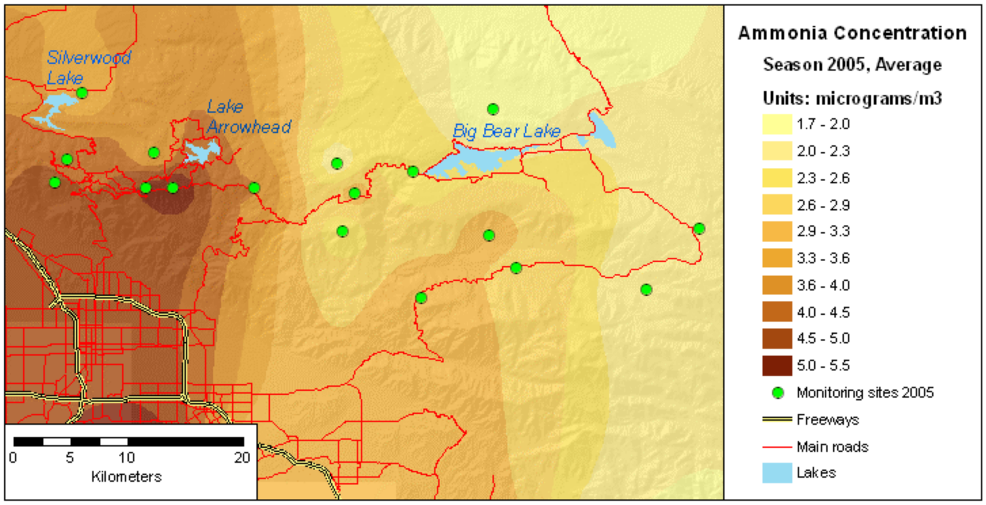

**FIGURE 5D**

Elevated levels of N deposition increase drought stress of trees, lead to build up of dead biomass in forest stands, and contribute to contamination of stream and surface water with nitrate[26]. These effects and those caused by elevated $O_3$ levels may cause deterioration of health of trees, change forest species composition, and predispose the SBM forests to widespread outbreaks of bark beetle and catastrophic fires, such as those which took place in the fall of 2003[27].

## ACKNOWLEDGMENTS

Study supported by the USDA Forest Service Pacific Southwest Research Station and USDA Forest Service Forest Health Monitoring Network. This research was partially funded by NSF grant DEB 04-21530 and USDA Forest Service Forest Health Monitoring Program.

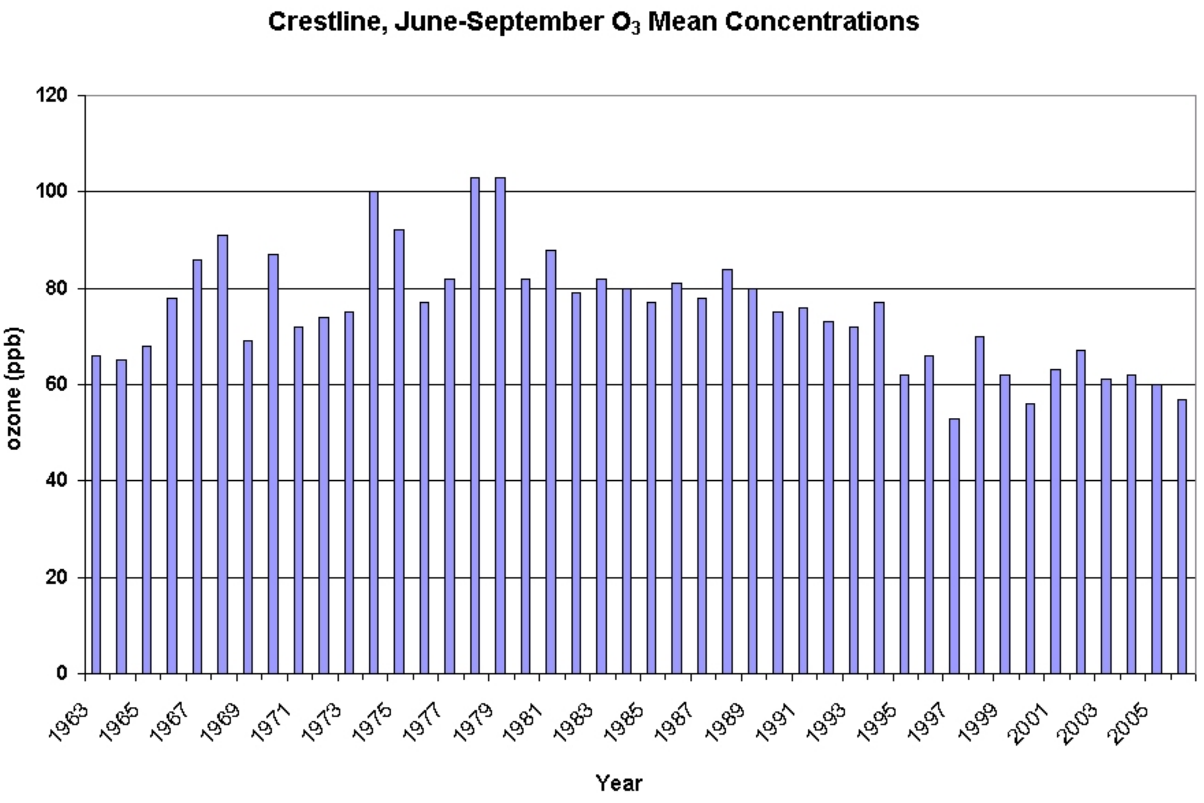

**FIGURE 6.** Changes in June–September mean 24-h $O_3$ concentrations at the Crestline/Camp site since 1963[14,15].
